# THE RELATIONSHIPS BETWEEN INFORMATION SOURCES, PERSPECTIVES ON PUBLIC HEALTH POLICIES, AND AGEISM

**DOI:** 10.1093/geroni/igae098.3259

**Published:** 2024-12-31

**Authors:** Cherrie Park

**Affiliations:** The Ohio State University, Columbus, Ohio, United States

## Abstract

Global concerns over heightening ageism have been raised throughout and after the pandemic. While it was argued that age-based public health policies during the pandemic contributed to intergenerational tension, little evidence was available about these concerns. In response, this study aimed to understand younger adults’ ageism in the context of public health crisis by examining the relationships between younger adults’ perspectives on health policies, sources of health information, and the intensity of their ageism. For a quantitative inquiry, survey data were collected in the United States from 317 individuals ages 18 to 44. Their ageism levels were measured with the Ambivalent Ageism Scale, which had been developed to capture multifaceted attitudes (i.e., hostile and benevolent) toward older adults. Multiple regression analysis revealed that benevolent ageism was more intense than hostile ageism among the survey respondents. The intensity of ageism was significantly associated with their beliefs about safety measures and the prioritization of older adults in distributing medical resources, as well as with their choice of information sources (e.g., social media) to receive health information. The findings inform people doing advocacy work for older adults, including those in the helping profession, about future directions for combating ageism in times of public health crisis. For example, they should guide people to utilize various information sources and accept health information critically. This study can be applied to developing effective education models, policies, and practices for alleviating ageism.

